# *Partner of bursicon* Regulates Pheromone Gland Development and Sex Pheromone Biosynthesis in *Helicoverpa armigera*

**DOI:** 10.3390/biom16081204

**Published:** 2026-08-18

**Authors:** Ziling Tang, Yuhao Liu, Huanhuan Zhang, Qing Zhai, Liuyi Fan, Xin Zhang, Du Li, Long Chen, Xiang Li

**Affiliations:** 1 Henan International Laboratory for Green Pest Control, College of Plant Protection, Henan Agricultural University, Zhengzhou 450046, China; tangziling4312@163.com (Z.T.); liuyuhao318@163.com (Y.L.); zhaiqing@henau.edu.cn (Q.Z.); fanliuyi522@126.com (L.F.); zhangxin_1104@163.com (X.Z.); lidu0215@163.com (D.L.); 2 Yunnan Key Laboratory of Tea Science, Tea Research Institute, Yunnan Academy of Agricultural Sciences, Kunming 650201, China; 3 Institute of Vegetables Research, Xizang Academy of Agriculture and Animal Husbandry Sciences, Lhasa 850032, China; xznkyzhh@163.com

**Keywords:** *Helicoverpa armigera*, bursicon, reproductive communication, mating behavior, oviposition

## Abstract

Bursicon, a heterodimeric neuropeptide composed of Burs-α and its binding subunit *Partner of bursicon* (*Pburs*), is primarily known to regulate insect cuticle tanning and also participates in wing expansion, reproduction and immunity. *Pburs* is highly expressed during pheromone gland (PG) maturation in female *Helicoverpa armigera*, a major agricultural pest, indicating its potential role in PG development and function. In this study, we cloned the full-length coding sequence of *HaPburs* and verified its high conservation across insects. qPCR revealed PG transcripts peaking at 48 h post-emergence in scotophase. RNAi-mediated knockdown reduced *HaPburs* transcript levels by 60.01%, resulting in abnormal PG morphology characterized by irregular melanized protrusions. Silencing *HaPburs* lowered the major sex pheromone component (*Z*)-11-hexadecenal by 42.66%, reduced male attraction and mating rates to 59.15% and 56.25% of controls, and markedly decreased cumulative fecundity. *HaPburs*-knockdown significantly downregulated the transcription of *HaPKA* and *HaACC*, two key genes involved in sex pheromone biosynthesis. These results suggest that *HaPburs* mediates PG morphogenesis and sex pheromone biosynthesis, further modulating sex pheromone-mediated reproductive communication in *H. armigera*. The study expands the known reproductive functions of bursicon and provides a promising molecular target for eco-friendly pest management.

## 1. Introduction

Bursicon is a heterodimeric neuropeptide hormone secreted by the insect central nervous system. It was first identified in the hemolymph of *Calliphora erythrocephala* by Fraenkel and Hsiao in 1965, and named for its essential role in regulating cuticle tanning [1]. The hormone comprises two subunits, bursicon α (Burs-α) and bursicon β (Burs-β), encoded by the *bursicon* (*burs*) and *Partner of bursicon* (*Pburs*) genes, respectively [2]. Both subunits are highly conserved across insect species, each containing 11 cysteine residues that form a characteristic cystine-knot motif through disulfide bonds, which is critical for maintaining protein structural stability [3]. While the Burs-α/Burs-β heterodimer functions as the canonical ligand mediating cuticle tanning, each subunit can also independently form homodimers with distinct biological roles [4,5].

LGR2 (leucine-rich repeat G protein-coupled receptor 2) belongs to the GPCR superfamily and contains an extracellular leucine-rich repeat domain. Its expression spans the insect epidermis, nervous system, and other functional organs [6]. Binding of bursicon to LGR2 triggers the cAMP-PKA signaling cascade, subsequently promoting dopamine biosynthesis [3,7,8]. Dopamine contributes to cuticle tanning through two complementary mechanisms: its derivatives reinforce the protein-chitin matrix via oxidative cross-linking to enhance cuticular mechanical strength, while dopamine is converted into insoluble melanin granules that deposit in the cuticle, causing cuticular darkening [9,10]. In *Drosophila melanogaster*, loss-of-function mutations in *burs* lead to defects in cuticle sclerotization and abnormal wing expansion in newly emerged adults [11]. Similarly, RNA interference (RNAi)-mediated knockdown of *HLGR2*, the bursicon receptor gene, in final-instar *Hyphantria cunea* larvae prevents pupal cuticle tanning and significantly increases mortality [12].

Beyond its canonical role in cuticle tanning, bursicon has been shown to participate in diverse physiological processes in insects, including wing expansion, reproduction, and innate immunity [5,13,14]. In *Drosophila melanogaster*, mutations in *burs* inhibit wing expansion and result in the persistence of intact wing epithelia within unexpanded wings [15]. RNAi-mediated knockdown of *burs*, *pburs*, or *rickets* in *Bemisia tabaci* significantly reduced juvenile hormone (JH) titers and suppressed the expression of vitellogenin (*Vg*) and its receptor (*VgR*), thereby impairing ovarian development and decreasing fecundity and egg hatchability [16]. Bursicon has also been implicated in insect immune defense. In *Aedes aegypti*, both Burs-α and Burs-β homodimers significantly induced the expression of antimicrobial peptide genes, including *Att*, *CecA*, and *DefA*, thereby enhancing host resistance to pathogenic infections [17].

*Helicoverpa armigera* is a globally important agricultural pest and a representative model for studies of insect physiology and behavior because of its wide distribution, broad host range, and strong destructive capacity [18]. Preliminary transcriptomic screening suggested that *HaPburs* expression increased during pheromone gland (PG) maturation in female *H. armigera*, indicating a potential role for *HaPburs* in PG development and function. In the present study, the full-length coding sequence of *HaPburs* was cloned and subjected to homology and phylogenetic analyses. RNAi was employed to characterize the regulatory functions of *HaPburs* in PG development and sex pheromone biosynthesis. Elucidation of these mechanisms will clarify the role of *Pburs* in insect pheromone communication and further expand current knowledge of bursicon-mediated regulatory networks in insects.

## 2. Materials and Methods

### 2.1. Insects

*H. armigera* specimens were originally collected from Jiyuan City, China. A laboratory colony was maintained for multiple generations under controlled conditions of 26 ± 1 °C, 75 ± 10% relative humidity, and a 16 h:8 h (L:D) photoperiod. Larvae were reared on an artificial diet, while adults received a 5% (*w*/*v*) honey solution as a nutritional supplement [19].

### 2.2. Target Gene Cloning

Total RNA was extracted from pooled adult and larval samples of *H. armigera* using the TransZol Up Plus RNA Kit (TransGen Biotech Co., Ltd., Beijing, China). First-strand cDNA was synthesized with HiScript III RT SuperMix (Vazyme Biotech Co., Ltd., Nanjing, China). *HaPburs* was amplified via PCR using KOD One PCR Master Mix (TOYOBO Co., Ltd., Osaka, Japan) under the following thermal cycling conditions: 95 °C for 5 min; 36 cycles of 95 °C for 30 s, 54 °C for 30 s, and 72 °C for 1 min; followed by a final extension at 72 °C for 10 min. Purified PCR products were cloned into the ZT4-Blunt vector using the ZT4-Blunt Fast Cloning Kit (Beijing Zoman Biotechnology Co., Ltd., Beijing, China). Recombinant plasmids were transformed into *Escherichia coli* DH5α competent cells and cultured at 37 °C for 12–16 h. Positive clones were identified through colony PCR and verified by Sanger sequencing (Tsingke Biotechnology Co., Ltd., Beijing, China). All qPCR primers were validated by melting curve analysis to ensure specific amplification. Primer sequences are provided in Table 1.

### 2.3. Phylogenetic Analysis

*Pburs* homologs were identified through BLAST searches (BLAST+ v2.17.0) against the NCBI database (https://blast.ncbi.nlm.nih.gov/Blast.cgi (accessed on 12 August 2026)). Multiple sequence alignment was performed using DNAMAN v.9.0, and conserved domains were predicted via InterProScan (http://www.ebi.ac.uk/interpro/ (accessed on 12 August 2026)). Phylogenetic reconstruction was conducted in MEGA v.12.0 using the Neighbor-Joining method with 1000 bootstrap replicates to assess node support [20].

### 2.4. Expression Analysis of HaPburs

To assess developmental expression dynamics of *HaPburs* in PG, PG samples were collected at 48 h pre-emergence, immediately after emergence (0 h), and 48 h post-emergence from virgin female moths. Total RNA was extracted from pooled samples prepared separately for each developmental stage for qPCR analysis. Quantitative real-time PCR (qPCR) was performed using Hieff qPCR SYBR Green Master Mix (Yeasen Biotechnology (Shanghai) Co., Ltd., Shanghai, China) on an Applied Biosystems 7500 Fast Real-Time PCR System (Applied Biosystems, Foster City, CA, USA). Expression levels were normalized using the 2^−ΔΔCt^ method, with *actin* (GenBank accession no. XM_021337727.3) and *EF1*α (XM_021329969.3) of *H. armigera* serving as internal reference genes [21]. Each tissue or developmental stage included three biological replicates, with each replicate consisting of 10 pooled PGs. Primer sequences are listed in Table 1.

### 2.5. RNA Interference (RNAi) Experiment

The target sequence of *HaPburs* was amplified from a mixed cDNA pool prepared from *H. armigera* adults and larvae using T7 promoter-linked primers. Double-stranded RNA (dsRNA) was synthesized in vitro with the T7 RNAi Transcription Kit (Vazyme Biotech Co., Ltd., Nanjing, China) following the manufacturer’s instructions. For RNAi treatment, female pupae (approximately 48 h pre-emergence) received a 4 μL abdominal microinjection containing 10 μg ds*HaPburs* (2.5 μg/μL). Newly emerged females were administered a second 10 μg dose of ds*HaPburs* and maintained in isolation to prevent mating. An equivalent amount of ds*EGFP* (U76561) was injected as a control. RNAi efficiency was evaluated in four randomly selected females at 48 h after the second injection. At the same time point, PG morphology was examined in 30 females per treatment across three biological replicates, with 10 females examined in each replicate. Females showing irregular protruding black spots on the PG surface were recorded as exhibiting abnormal PG morphology, and the abnormality rate was calculated as the percentage of abnormal females among the total examined females. During the second scotophase post-emergence (2 h into the dark period), PGs were dissected for gene expression analysis of key pheromonogenesis-related genes, including *Pheromone Biosynthesis Activating Neuropeptide Receptor* (*PBANR*), *Calcineurin* (*CaN*), *Acetyl-CoA Carboxylase* (*ACC*), *Protein Kinase A* (*PKA*), *Fatty Acyl-CoA Reductase 2* (*FAR2*), and *Desaturase 11* (*Des11*). Each treatment included three biological replicates, with each replicate consisting of 10 PGs.

### 2.6. Measurement of Pheromone Component Levels in PGs

The sex pheromone components (*Z*)-11-hexadecenal (*Z*11-16:Ald) and (*Z*)-9-hexadecenal (*Z*9-16:Ald) were extracted from dissected PGs of 48 h post-emergence virgin female *H. armigera* collected 4 h after the onset of the scotophase, using 200 μL of *n*-hexane (>99% purity) for 24 h. Relative comparisons were performed based on GC-FID peak areas according to previously reported methods [22,23,24]. Aliquots of the extract (2 μL) were injected in splitless mode into an Agilent 7890A gas chromatograph (Agilent Technologies, Santa Clara, CA, USA) equipped with an HP-5 capillary column (30 m × 0.32 mm i.d. × 0.25 μm film thickness) and a flame ionization detector (FID) operated at 260 °C. Nitrogen was used as the carrier gas at a constant column flow rate of 0.75 mL/min and as the FID make-up gas at 25 mL/min. The oven temperature was increased from 150 °C to 230 °C at 30 °C/min and then to 260 °C at 20 °C/min. Target peaks were assigned by retention-time matching with authentic standards under identical GC-FID conditions; without an internal standard or external calibration curve, peak areas were normalized to the corresponding ds*EGFP* mean (100%) for relative comparison only. Each treatment consisted of three biological replicates, with each sample containing 10 PGs.

### 2.7. Behavioral Assay for Male Attraction

Female pupae (48 h pre-emergence) were microinjected with 10 μg of ds*HaPburs* into the posterior abdominal segment. On the morning of emergence, newly emerged females received a second injection of 10 μg ds*HaPburs* and were maintained individually to ensure virginity. Females injected with an equivalent amount of ds*EGFP* served as the RNAi negative control, whereas females injected with an equivalent volume of RNase-free water served as the vehicle control. Twenty treated virgin females (48 h post-emergence) were randomly assigned to a trapping chamber, while 20 ds*EGFP*-treated control virgin females of the same age were placed into a second identical chamber. The positions of the treatment and control chambers were randomized in each experimental replicate. Each chamber measured 50 × 50 × 50 cm and was equipped with a ventilation system providing an airflow rate of 1.5 L/min. A separate release chamber containing 40 experimentally naïve 2–4-day-old virgin males was used to evaluate changes in male attraction toward ds*HaPburs*-treated females. Each male was used in only one behavioral trial. Trapping assays were conducted under dark conditions at 26 °C, and the number of males captured in each chamber was recorded at 06:00 h the following morning. Each treatment included three biological replicates.

### 2.8. Mating Rate Assay

At 48 h pre-emergence, female pupae of *H. armigera* received an abdominal microinjection of 10 μg ds*HaPburs*, followed by a second injection in newly emerged adults to reinforce RNAi. Control individuals were injected with an equivalent amount of ds*EGFP*. Twenty treated females and 20 virgin males were placed in a 50 × 50 × 50 cm mating cage maintained in complete darkness at 26 °C. At 06:00 h the following morning, females were dissected to examine spermatophores for mating confirmation. Each treatment consisted of three biological replicates. Within each biological replicate, the ds*HaPburs* and ds*EGFP* groups were conducted simultaneously using insects from the same experimental cohort under identical environmental conditions.

### 2.9. Oviposition Behavior Assay

At 48 h pre-emergence, female pupae of *H. armigera* received an abdominal microinjection of 10 μg ds*HaPburs*, followed by a second injection in newly emerged adults and a third injection at 48 h post-emergence to reinforce RNAi. Control individuals received an equivalent amount of ds*EGFP*. For oviposition assessment, each treated female was paired with two virgin males in a custom-designed oviposition cage. Egg production was monitored daily. Each treatment included nine biological replicates, with one female per replicate.

### 2.10. Data Analysis

Statistical analyses were performed using IBM SPSS Statistics 23.0. All data are presented as mean ± SEM. For the spatiotemporal expression analysis, *HaPburs* expression levels among three developmental stages of PGs collected at 48 h pre-emergence, 0 h post-emergence, and 48 h post-emergence, were analyzed together using one-way ANOVA followed by Tukey’s multiple comparison test. Differences between ds*HaPburs* and ds*EGFP* treatments in RNAi efficiency, the expression levels of sex pheromone biosynthesis-related genes, relative levels of pheromone components extracted from PGs, mating rate, and oviposition parameters were analyzed using Student’s *t*-tests. For the two pheromone-associated components, the resulting *p* values were adjusted using the Holm procedure. Daily egg counts were compared between treatments separately within each day using two-sided unpaired Student’s *t*-tests, with Holm adjustment across the 10 day-specific comparisons. Male attraction and mating frequency were analyzed using paired Student’s *t*-tests because the ds*HaPburs* and ds*EGFP* groups were conducted simultaneously and matched within each biological replicate. Statistical significance was defined at *p* < 0.05.

## 3. Results

### 3.1. Sequence Analysis of HaPburs in H. armigera

**Figure 1:**
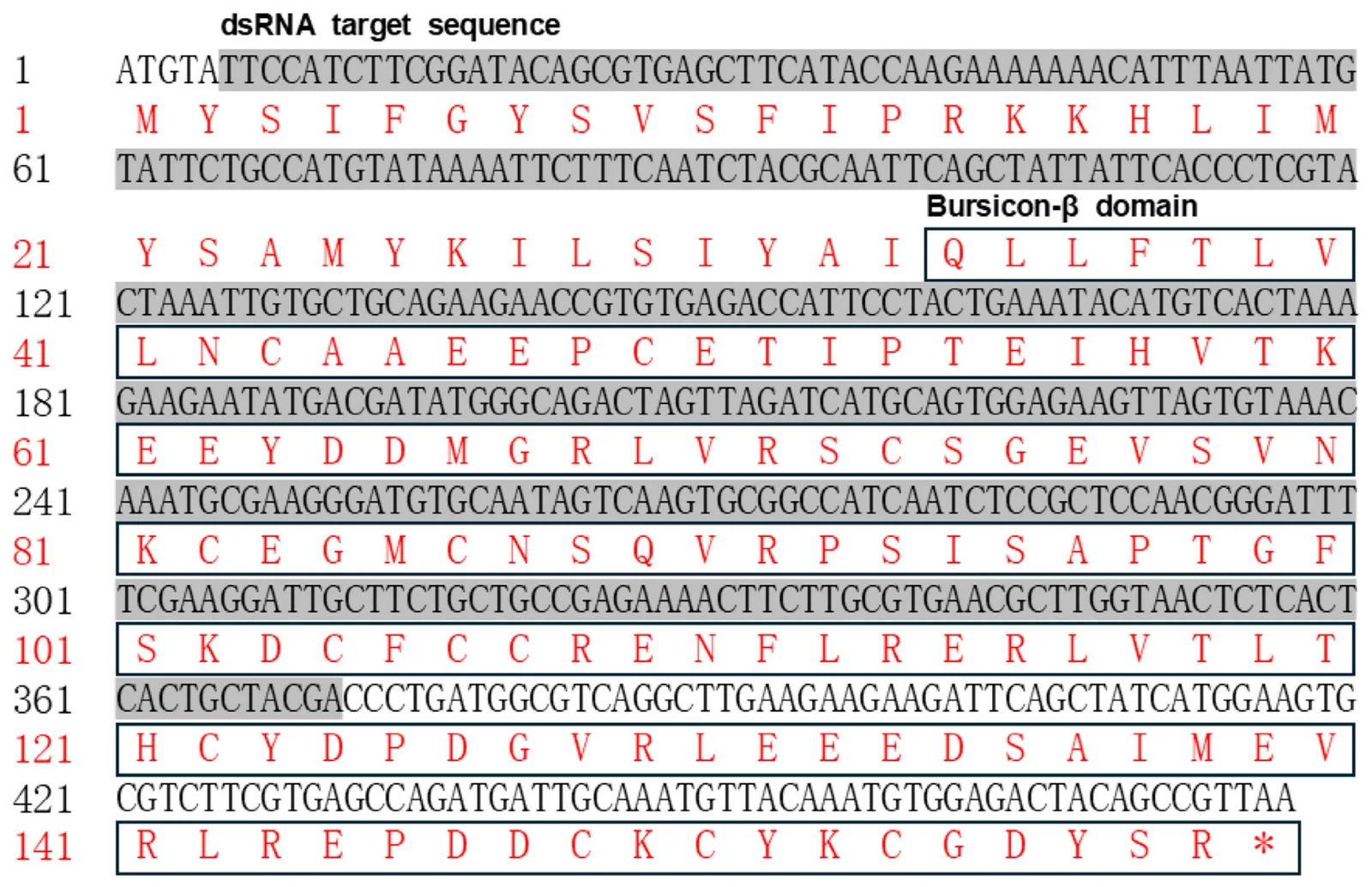
Nucleotide and deduced amino acid sequences of the Burs-β subunit in *Helicoverpa armigera*. Black letters and numbers indicate the nucleotide sequence and nucleotide positions, respectively; red letters and numbers indicate the deduced amino acid sequence and amino acid positions, respectively. The asterisk denotes the stop codon. The gray-shaded region indicates the ds*HaPburs* target sequence, and the boxed region denotes the Burs-β subunit domain.

The *HaPburs* transcript contains a 477 bp open reading frame encoding a protein of 158 amino acids. The deduced Burs-β protein has a predicted molecular weight of 18.15 kDa and a theoretical isoelectric point of 5.27. Structural analysis identified a typical bursicon β-subunit domain located at residues 34–158 (Figure 1).

**Figure 2:**
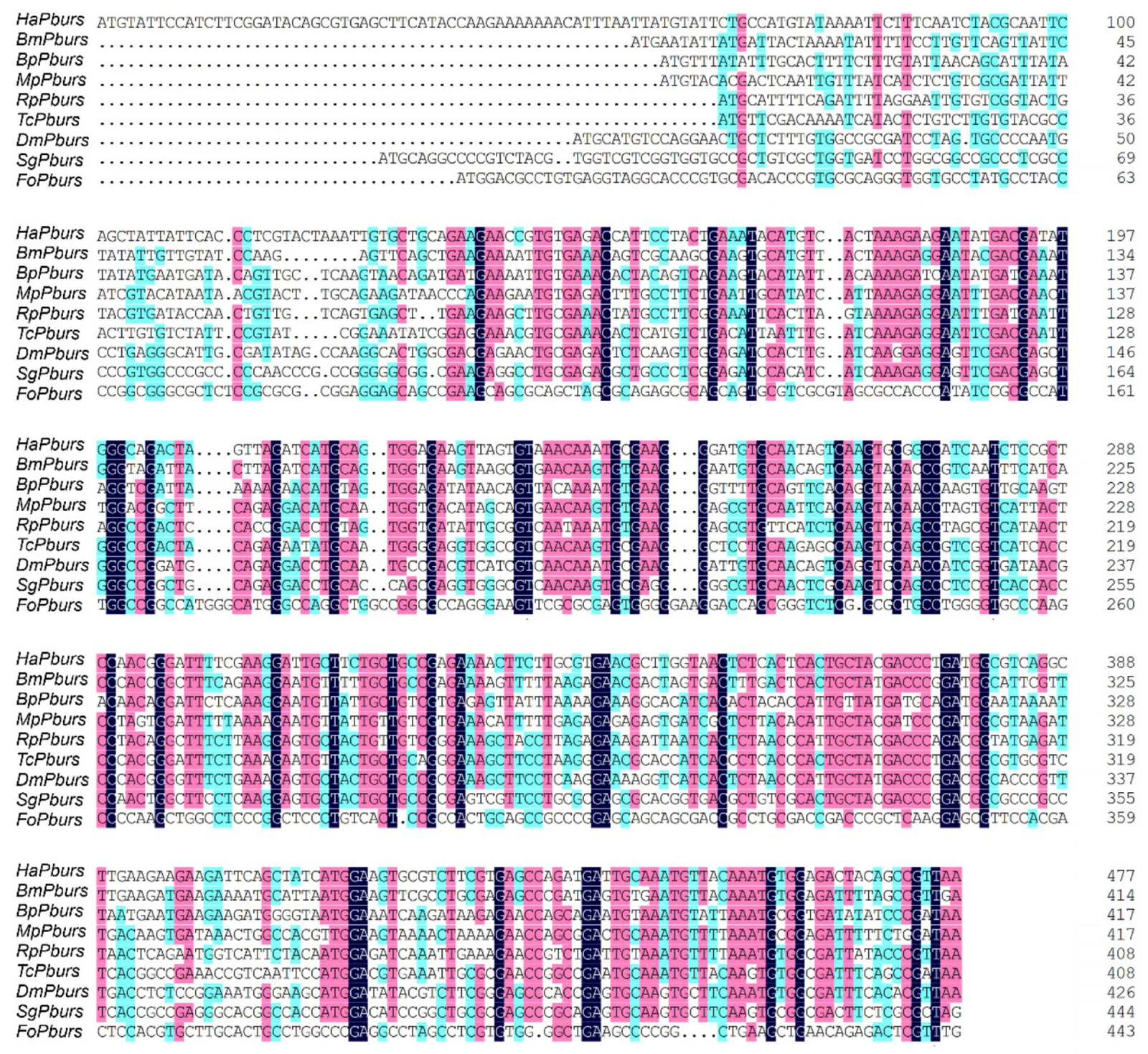
Multiple sequence alignment of *Pburs* nucleotide sequences from *Helicoverpa armigera* and eight other insect species. Species and GenBank Accession Numbers: *HaPburs*: *H. armigera*, XM_021326060.3; *BmPburs*: *Bombyx mori*, NM_001043824.1; *BpPburs*: *Bombus pascuorum*, XM_060957664.1; *MpPburs*: *Myzus persicae*, XM_022316017.1; *RpPburs*: *Rhodnius prolixus*, XM_074120606.1; *TcPburs*: *Tribolium castaneum*, NM_001114308.1; *DmPburs*: *Drosophila melanogaster*, NM_135868.2; *SgPburs*: *Schistocerca gregaria*, XM_049987824.1; *FoPburs*: *Frankliniella occidentalis*, XM_026424660.2. Sequence conservation is indicated by color shading: dark blue, 100% identity; red, 75–90% identity; light blue, 50–75% identity; and white, less than 50% identity.

Multiple nucleotide sequence alignment demonstrated that *HaPburs* shared the highest identity with *Bombyx mori* (62.89%) and the lowest identity with *Frankliniella occidentalis* (32.29%). Sequence identities between *HaPburs* and homologs from *Bombus pascuorum*, *Myzus persicae*, *Rhodnius prolixus*, *Tribolium castaneum*, *Drosophila melanogaster*, and *Schistocerca gregaria* ranged from 49.69% to 53.77%, indicating moderate to high conservation of *Pburs* among different insect lineages (Figure 2).

### 3.2. Phylogenetic Analysis of Bursicon β Subunit in Insects

**Figure 3:**
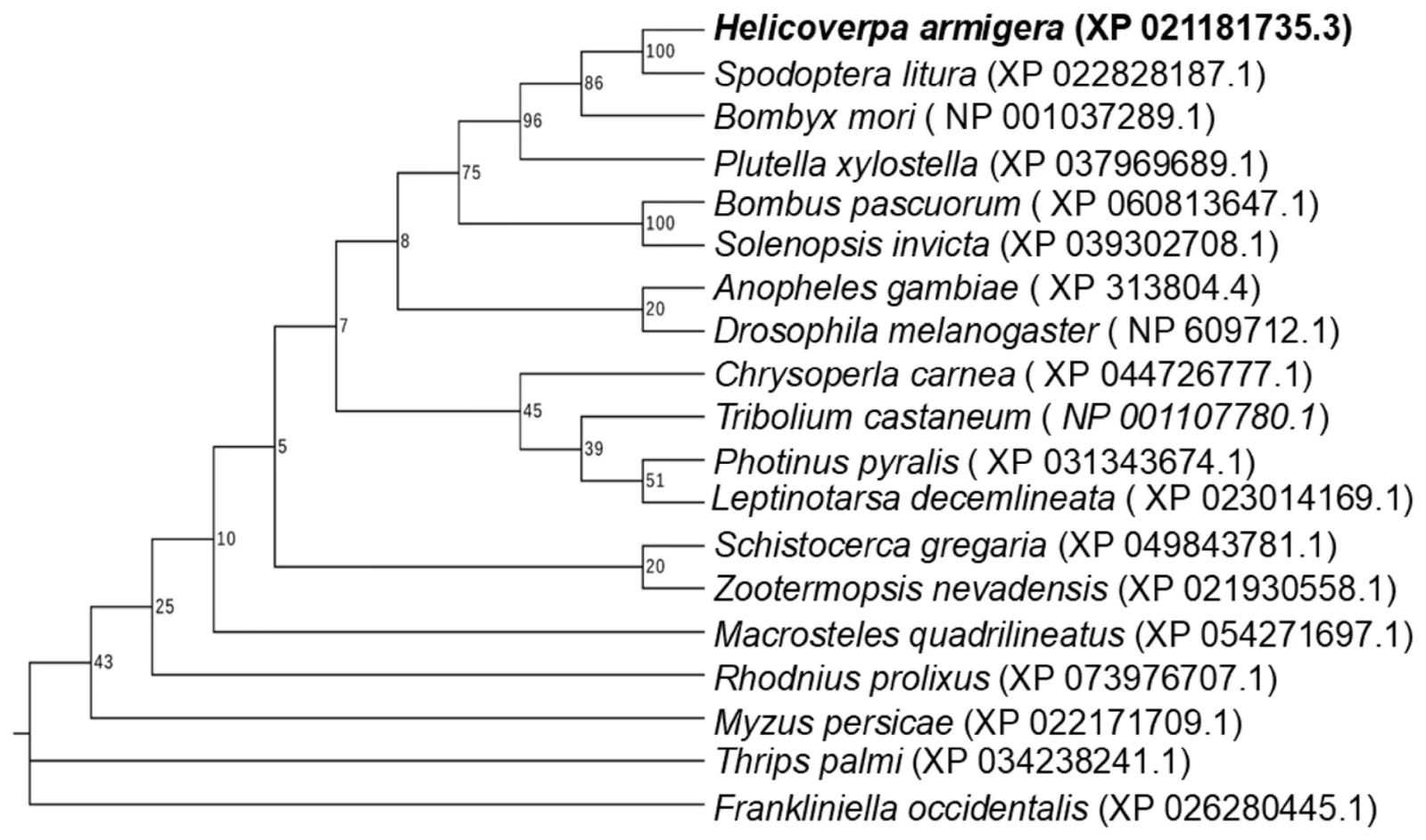
Phylogenetic relationships of bursicon β subunits among diverse insect species. Species and GenBank accession numbers: *Helicoverpa armigera* (XP_021181735.3), *Spodoptera litura* (XP_022828187.1), *Bombyx mori* (NP_001037289.1), *Plutella xylostella* (XP_037969689.1), *Bombus pascuorum* (XP_060813647.1), *Solenopsis invicta* (XP_039302708.1), *Anopheles gambiae* (XP_313804.4), *Drosophila melanogaster* (NP_609712.1), *Chrysoperla carnea* (XP_044726777.1), *Tribolium castaneum* (NP_001107780.1), *Photinus pyralis* (XP_031343674.1), *Leptinotarsa decemlineata* (XP_023014169.1), *Schistocerca gregaria* (XP_049843781.1), *Zootermopsis nevadensis* (XP_021930558.1), *Macrosteles quadrilineatus* (XP_054271697.1), *Rhodnius prolixus* (XP_073976707.1), *Myzus persicae* (XP_022171709.1), *Thrips palmi* (XP_034238241.1), *Frankliniella occidentalis* (XP_026280445.1). All amino acid sequences analyzed were retrieved from the NCBI database. The phylogenetic tree was constructed using the neighbor-joining method in MEGA v.12.0. Bootstrap analysis was performed with 1000 replicates to evaluate support for the clustering of associated taxa. Percentages above the branches indicate the frequency with which taxa clustered together in replicate trees.

Phylogenetic analysis showed that the Burs-β sequence of *H. armigera* clustered closely with that of *Spodoptera litura*, consistent with their taxonomic classification within the same family. These two noctuid species further grouped with *Bombyx mori* and *Plutella xylostella* to form a Lepidoptera-specific clade supported by a high bootstrap value (96%), indicating strong evolutionary conservation of Burs-β within this order. In addition, Burs-β sequences from other insect orders, including Hymenoptera, Coleoptera, Diptera, Hemiptera, and Orthoptera, formed distinct and well-separated clades, reflecting order-specific evolutionary patterns of the Burs-β subunit. Bootstrap support for deeper nodes separating the major insect orders remained relatively low (Figure 3).

### 3.3. Expression Pattern of HaPburs in PG of Female H. armigera

**Figure 4:**
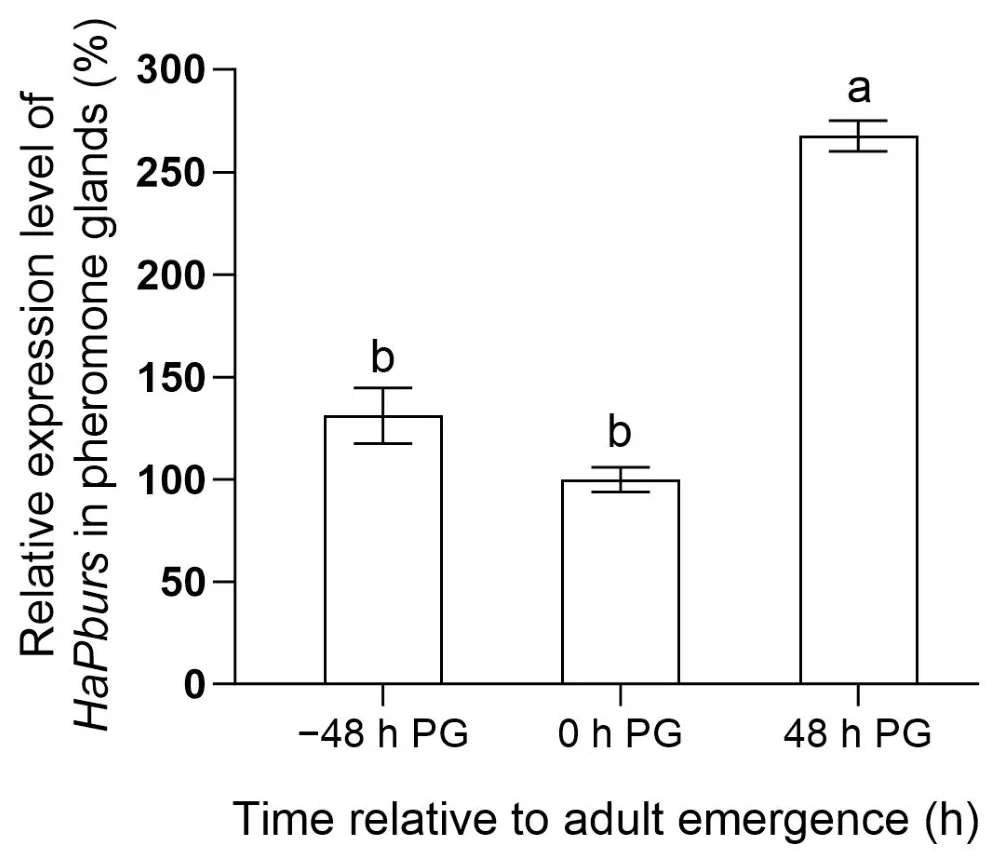
Developmental expression patterns of *HaPburs* in pheromone glands (PG) of female *Helicoverpa armigera*. Data are presented as mean ± SEM of three biological replicates, with each replicate consisting of 10 PGs. Different letters above the bars indicate significant differences among the indicated groups (*p* < 0.05).

qPCR analysis detected significant dynamic changes in *HaPburs* transcripts during PG maturation. The expression level of *HaPburs* in PG reached the maximum at 48 h post-emergence, which was significantly 2.04- and 2.68-fold higher than the levels at 48 h pre-emergence and 0 h post-emergence, respectively (*p* = 0.00014 and 0.000043, respectively; Figure 4).

### 3.4. Effects of HaPburs Knockdown on PG Morphology, Pheromone Production, Reproductive Behavior, and Fecundity in Female H. armigera

**Figure 5:**
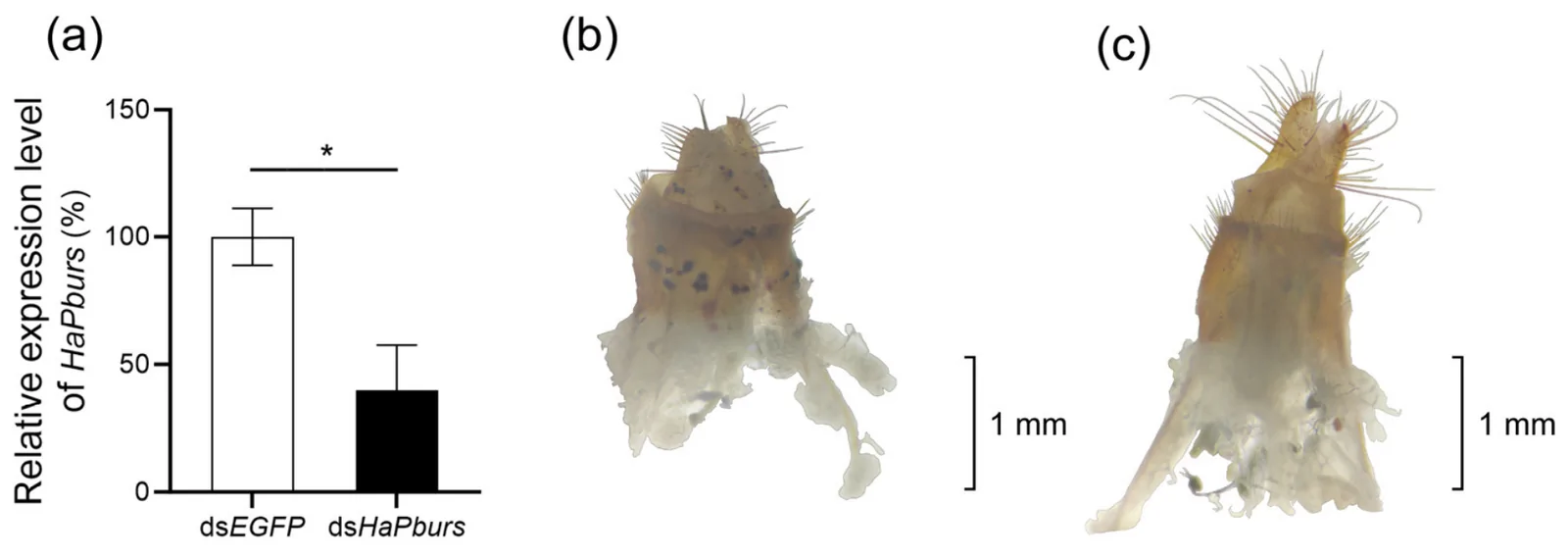
Effects of *HaPburs*-RNAi on pheromone gland morphology in female *Helicoverpa armigera*. (**a**) Knockdown efficiency of *HaPburs* in the pheromone glands (PGs) of adult females following ds*HaPburs* injection. Females injected with ds*EGFP* served as controls. Data are presented as mean ± SEM of four biological replicates. Asterisks indicate significant differences compared with the ds*EGFP* control according to Student’s *t*-test (* *p* < 0.05). (**b**) Representative PG phenotype of ds*HaPburs*-injected females showing irregular melanized protrusions. (**c**) Representative PG phenotype of ds*EGFP*-injected females. Scale bars in (**b**,**c**) = 1 mm.

Following ds*HaPburs*-mediated RNAi, *HaPburs* transcript levels decreased significantly by approximately 60.01% compared with the ds*EGFP* group (*p* = 0.0157; Figure 5a). Concurrently, morphological examination showed that 76.67% of ds*HaPburs*-treated females exhibited irregular protruding black spots on the PG surface (Figure 5b,c). Notably, this melanized abnormality was not observed in any of the ds*EGFP* control individuals, nor in untreated wild-type female moths dissected under identical culture conditions.

**Figure 6:**
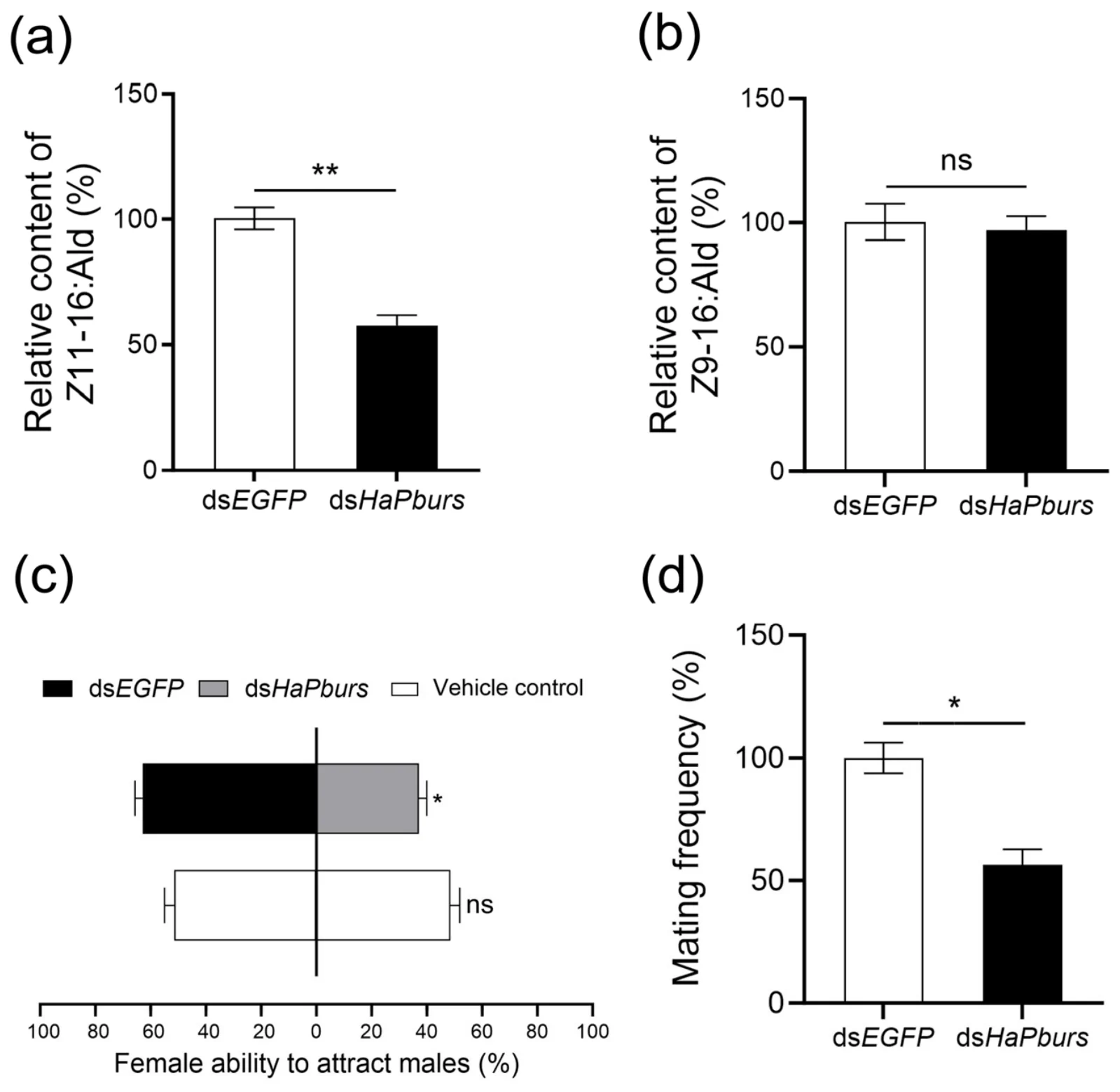
Effects of *HaPburs*-RNAi on sex pheromone component levels and reproductive behavior in *Helicoverpa armigera*. (**a**) Relative content of *Z*11-16:Ald in female pheromone glands (PGs) following *HaPburs*-RNAi. (**b**) Relative content of *Z*9-16:Ald in female PGs following *HaPburs*-RNAi. For (**a**,**b**), each biological replicate consisted of 10 PGs. (**c**) Effects of female *HaPburs*-RNAi on male attraction to treated females. Females injected with an equivalent amount of ds*EGFP* served as the RNAi negative control, whereas females injected with an equivalent volume of RNase-free water served as the vehicle control. Each replicate included 20 females per treatment and 40 virgin males for attraction assessment. (**d**) Mating frequency of ds*HaPburs*-treated and ds*EGFP*-treated females. Each replicate included 20 female–male pairs per treatment. Data are shown as mean ± SEM from three independent biological replicates. Asterisks indicate significant differences between treatments within each panel (Student’s *t*-test: * *p* < 0.05, ** *p* < 0.01, ns, not significant).

*HaPburs*-RNAi significantly reduced the content of the major sex pheromone component *Z*11-16:Ald by 42.66% relative to the control group (*p* = 0.0034; Figure 6a), whereas the minor component *Z*9-16:Ald did not change significantly (*p* = 0.7698; Figure 6b). Male attraction and mating rates decreased to 59.15% and 56.25% of the corresponding control values, respectively (*p* = 0.0413 and 0.0261, respectively; Figure 6c,d).

**Figure 7:**
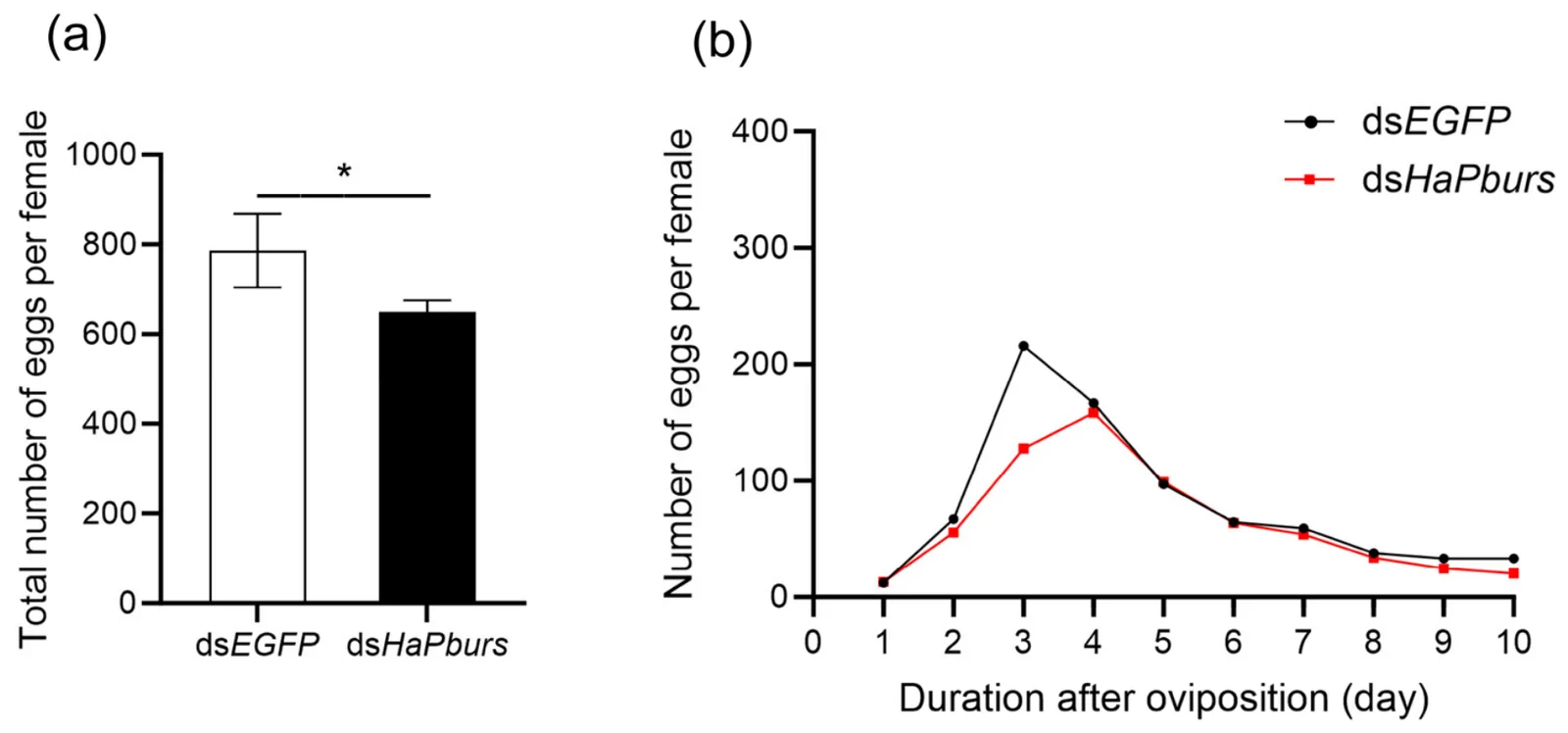
Effects of *HaPburs*-RNAi on total and daily fecundity in female *Helicoverpa armigera*. (**a**) Total egg production. Groups were compared using a two-sided unpaired Student’s *t*-test (*p* = 0.0128). (**b**) Daily egg production. Treatment groups were compared separately within each day using two-sided unpaired Student’s *t*-tests, and the resulting *p* values were adjusted across the 10 comparisons using the Holm procedure. Significant differences were detected on Days 3 and 10 (adjusted *p* = 0.0169 and 0.0386, respectively). Data are presented as mean ± SEM of nine biological replicates, with one female per replicate. The asterisk in (**a**) indicates a significant difference between treatments (*p* < 0.05).

Furthermore, ds*HaPburs* injection significantly reduced cumulative egg production per female by 137 eggs compared with the ds*EGFP* control (*p* = 0.0128; Figure 7a). Daily egg production differed significantly between treatments on Days 3 and 10 after Holm adjustment across the 10 day-specific comparisons (adjusted *p* = 0.0169 and 0.0386, respectively), whereas no significant differences were detected on the other days (Figure 7b).

### 3.5. Effects of HaPburs Knockdown on Genes Involved in the Sex Pheromone Biosynthesis Pathway in Female H. armigera

**Figure 8:**
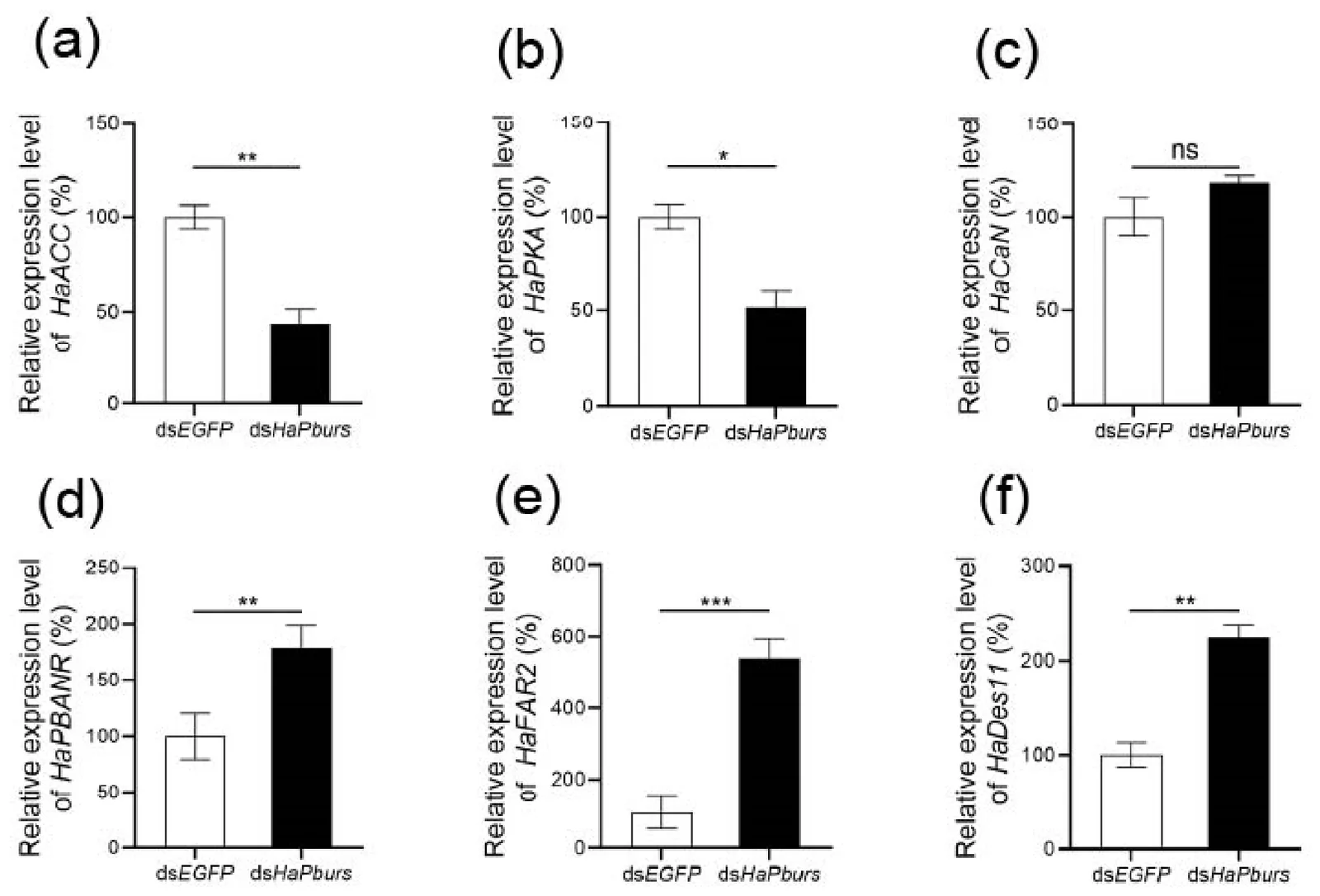
Effects of *HaPburs*-RNAi on the expression of pheromone biosynthesis-related genes in female *Helicoverpa armigera*. (**a**) *HaACC*, (**b**) *HaPKA*, (**c**) *HaCaN*, (**d**) *HaPBANR*, (**e**) *HaFAR2*, and (**f**) *HaDes11*. Gene expression was measured in pheromone glands (PGs) of ds*HaPburs*- and ds*EGFP*-treated females. Females injected with ds*EGFP* served as controls. Data are presented as mean ± SEM of three biological replicates, with each replicate consisting of 10 pooled PGs. *Actin* and *EF1α* were used as reference genes for normalization. Asterisks indicate significant differences between treatments within each panel (Student’s *t*-test: * *p* < 0.05, ** *p* < 0.01, *** *p* < 0.001; ns, not significant).

Following *HaPburs* knockdown, transcript levels of *HaACC* and *HaPKA* decreased by 56.07% and 48.44%, respectively, relative to the control (*p* = 0.0052 and *p* = 0.0107, respectively; Figure 8a,b). In contrast, *HaCaN* expression remained unaffected (*p* = 0.1558; Figure 8c), whereas *HaPBANR*, *HaFAR2*, and *HaDes11* expression levels were significantly upregulated to varying degrees (*p* = 0.0084, *p* = 0.0004, and *p* = 0.0022, respectively; Figure 8d–f).

## 4. Discussion

Bursicon is a key neurohormone involved in insect cuticle tanning [1]. This process contributes to exoskeletal morphogenesis and structural integrity by regulating dopamine metabolism and the cross-linking of cuticular proteins [9,10]. Accumulating evidence has demonstrated that bursicon exerts pleiotropic functions beyond classical cuticle tanning, including wing expansion, ovarian development, and innate immunity [5,8,25], indicating its broad regulatory potential within the neuroendocrine signaling network. In the present study, the regulatory roles of the *HaPburs* in PG development and sex pheromone biosynthesis in female *H. armigera* adults were investigated from the perspective of sex pheromone-mediated reproductive communication. These findings expand the functional repertoire of bursicon and provide a potential molecular basis for the development of reproductive disruption-based pest management strategies.

*HaPburs* exhibited high conservation within the bursicon β domain across insect *Pburs* orthologs, suggesting the retention of conserved physiological functions. Notably, the developmental variation in *HaPburs* transcript abundance detected in pooled PG preparations suggests a potential association with PG function and PG-mediated reproductive communication, particularly in sex pheromone biosynthesis and release. Supporting this inference, Li et al. [25] reported that bursicon activates the IIS/TOR and JH signaling pathways via the receptor *rickets*, thereby promoting *Vg* synthesis and ovarian development in *Tribolium castaneum*. Similarly, Yu et al. [16] demonstrated that RNAi-mediated silencing of *burs*, *pburs*, or *rickets* significantly reduced JH titers and *Kr-h1* expression, suppressed transcription of *Vg* and its receptor, and ultimately resulted in impaired ovarian development, decreased fecundity, and reduced egg hatchability. Collectively, these studies provide indirect experimental evidence supporting the involvement of *HaPburs* in reproductive regulation in *H. armigera*.

RNAi-mediated suppression of *HaPburs* significantly reduced the level of *Z*11-16:Ald, the major sex pheromone component in *H. armigera*, indicating that *HaPburs* participates in the regulation of sex pheromone biosynthesis and/or accumulation in the PG. Sex pheromones function as critical chemical signals in insect reproductive communication, and their titer and blend ratio are key determinants of female attractiveness to males [22,26,27]. The marked reductions in male attraction and mating success following *HaPburs* silencing were likely associated with the reduced *Z*11-16:Ald level, because pheromone titer and blend ratio are key determinants of male attraction in moths [22,26,27]. To our knowledge, this study provides experimental evidence linking bursicon signaling to the regulation of insect sex pheromone production. Previous studies mainly focused on the effects of bursicon on oviposition through regulation of *Vg*-related genes [16,25]. In contrast, the present results extend its functional scope to the early stages of pheromones communication, providing new insights into the multilevel regulatory roles of bursicon within the insect reproductive network.

Notably, 76.67% of *H. armigera* females exhibited irregular melanized protrusions on the PG surface following *HaPburs* knockdown. As a vital exocrine tissue responsible for sex pheromone biosynthesis and release, normal physiological function of the PG relies on intact biosynthetic pathways as well as complete tissue architecture supporting pheromone secretion, storage, and release. Exocrine glands function as compartmentalized dynamic secretory systems, and sustained structural integrity is a prerequisite for precise control of secretion rhythms [28]. Therefore, the morphological defects observed in *HaPburs*-silenced PGs may disrupt the integrity of secretory epithelial cells, disturb neuroendocrine signaling and intracellular substance transport, and ultimately hinder the efficient release of sex pheromones. Given the relatively short coding sequence of *HaPburs*, we were unable to design a second independent dsRNA fragment to further validate the specificity of RNAi phenotypes. In future studies, gene rescue experiments or tissue-specific RNAi could be performed to more precisely characterize the physiological function of this gene. Because only one dsRNA fragment was evaluated and neither rescue nor tissue-specific manipulation was performed, sequence-dependent off-target effects and indirect systemic effects cannot be fully excluded. It should be noted that the morphological characterization of PG lesions in this study was limited to macroscopic observation under a stereomicroscope. Histological staining and transmission electron microscopy were not applied to quantify the severity of melanotic lesions or resolve ultrastructural damage at the subcellular level. Follow-up quantitative histological assays will enable more accurate assessment of the severity of PG malformation triggered by *HaPburs* knockdown. Moreover, the exact molecular and cellular cascades through which *HaPburs* loss disrupts PG tissue structure remain to be further elucidated.

Previous studies demonstrated that bursicon binds to its receptor LGR2 and activates the cAMP-PKA signaling pathway to regulate diverse downstream physiological processes [3,7,8]. Our transcriptional data showed that silencing *HaPburs* reduced the expression of *PKA* and *ACC*, two core genes involved in sex pheromone biosynthesis [23,29,30]. This transcriptional correlation provides a preliminary indication of potential links between *HaPburs* and the cAMP-PKA-ACC pathway, yet we lack direct evidence including cAMP concentration and PKA phosphorylation detection to confirm the activation status of this signaling cascade. Further pharmacological or protein activity experiments will be required to validate this regulatory relationship. The decrease in sex pheromone production can be mainly attributed to the downregulation of *ACC*, a rate-limiting enzyme in fatty acid biosynthesis. Insufficient fatty acyl precursor supply directly restricts the overall synthesis and release of sex pheromones [23,29,30]. Although *HaFAR2* and *HaDes11* were upregulated after *HaPburs* knockdown, these two genes function in the downstream modification of fatty acyl chains and are not limited to sex pheromone biosynthesis [22,31,32]. They also participate in general lipid metabolism and other cellular processes. Since their catalytic roles depend on sufficient upstream precursors, their expression changes are not linearly correlated with final pheromone titer.

## 5. Conclusions

In conclusion, this study demonstrates that *Pburs* contributes to PG morphogenesis in *H. armigera* and modulates sex pheromone biosynthesis, thereby participating in reproductive communication. These findings expand the functional repertoire of bursicon and refine its regulatory network in insect reproduction. Moreover, because *Pburs* silencing significantly reduces mating and oviposition rates in *H. armigera*, this pathway represents a promising target for the development of eco-friendly pest management strategies aimed at disrupting the reproductive cycle of insect pests.

## Figures and Tables

**Table 1:** Primer sequences used in this study.

Name of Primer	GenBank No.	Amplification Efficiency (%)	Sequence of Primer (5′ → 3′)
**PCR primer**			
*Partner of bursicon* (*Pburs*)	XM_021326060.3		F: TTCCATCTTCGGATACAGCG
			R: TTGCTCCACACAACATAACG
**qPCR primers**			
*Actin*	XM_021337727.3	95.6	F: CCTGGTATTGCTGACCGTATGC
			R: CTGTTGGAAGGTGGAGAGGGAA
*Elongation factor 1 alpha* (*EF1α*)	XM_021329969.3	91.6	F: GAAGTCAAGTCCGTGGAGATG
			R: GACCTGTGCTGTGAAGTCG
*Partner of bursicon* (*Pburs*)	XM_021326060.3	93.5	F: GCTCATTTGTGCCTGTGTAG
			R: GCTCCACACAACATAACGATT
*PBAN receptor* (*PBANR*)	XM_064037567.1	99.8	F: CGTGATGAACACGACGAACG
			R: AGGTAAAAGTTGGTGGCGGT
*Calcineurin* (*CaN*)	XM_021325538.3	99	F: AAGATGCTGGTTACCGAATGT
			R: AAGGGACCAGGTAAAGACAT
*Acetyl-CoA carboxylase* (*ACC*)	XM_064042611.1	101.0	F: TCCTTCCGTCAACTCGCTCAG
			R: CTGCCTGTATCCGTTCTGAT
*Protein kinase A* (*PKA*)	XM_021330466.3	103.0	F: GCATAACCAGCCGCCATT
			R: ACCTCAAGCCCGAGAACC
*Fatty acyl-CoA reductase 2* (*FAR2*)	XM_064042714.1	102.0	F: CTTGTTGCACGTGCTGAGCT
			R: TGCACGAGCTGCAGTTGTAC
*Desaturase 11* (*Des11*)	XM_021327953.3	96.7	F: TGGGGCAACAAGCCTTATGA
			R: TTCAAGTCGTACGCCCATCC
**dsRNA primers**			
ds*HaPburs*	XM_021326060.3		F: T7+TTCCATCTTCGGATACAGCG
			R: T7+TCGTAGCAGTGAGTGAGAGT
ds*EGFP*	U76561.1		F: T7+ATCCTGTTACCAGTGGCTGC
			R: T7+CCGCTTACCGGATACCTGTC

T7 sequence: GGATCCTAATACGACTCACTATAGG.

## Data Availability

The data that support the findings of this study are available from the corresponding authors upon reasonable request.
